# Prevalence and Characterization of *Staphylococcus aureus* Isolated From Women and Children in Guangzhou, China

**DOI:** 10.3389/fmicb.2018.02790

**Published:** 2018-11-16

**Authors:** Bingshao Liang, Jialiang Mai, Yunfeng Liu, Yanmei Huang, Huamin Zhong, Yongqiang Xie, Qiulian Deng, Lianfen Huang, Shuwen Yao, Yanming He, Yan Long, Yiyu Yang, Sitang Gong, Hongling Yang, Zhenwen Zhou

**Affiliations:** ^1^Clinical Laboratory, Guangzhou Women and Children’s Medical Center, Guangzhou Medical University, Guangzhou, China; ^2^Clinical Laboratory, Zengcheng Maternity and Children’s Health Care Center, Guangzhou Medical University, Guangzhou, China; ^3^Clinical Laboratory, Guangzhou Maternity and Children’s Health Care Center, Guangzhou Medical University, Guangzhou, China; ^4^Clinical Laboratory, Nansha Maternity and Children’s Health Care Center, Guangzhou Medical University, Guangzhou, China; ^5^Pediatric Intensive Care Unit, Guangzhou Women and Children’s Medical Center, Guangzhou Medical University, Guangzhou, China; ^6^Department of Gastroenterology, Guangzhou Women and Children’s Medical Center, Guangzhou Medical University, Guangzhou, China

**Keywords:** epidemic clones, virulent genes, antibiogram, *Staphylococcus aureus*, MSSA, MRSA, *rpoB*

## Abstract

The prevalent *Staphylococcus aureus* clones and antibiotic susceptibility profiles are known to change dynamically and geographically; however, recent *S. aureus* strains causing infections in women and children in China have not been characterized. In this study, we analyzed the molecular epidemiology and antimicrobial resistance of *S. aureus* isolated from patients in four centers for women and children in Guangzhou, China. In total, 131 *S. aureus* isolates (100 from children and 31 from women) were analyzed by spa typing, multi-locus sequence typing, virulence gene and antimicrobial resistance profiling, staphylococcal chromosomal cassette *mec* typing, and mutation analyses of *rpoB*. A total of 58 spa types, 27 sequence types (STs), and 10 clonal complexes (CCs) were identified. While CC59 (ST59-IV, 48.8%; ST338-III, 35.7%) and CC45 (ST45-IV, 100%) were the major clones (84.4%) among MRSA isolates, CC5 (ST188, 24.3%; ST1, 21.6%) and CC398 (ST398, 70%) were the major ones (70.1%) among MSSA isolates. ST338-MRSA-III mostly found in pus but hardly in respiratory tract samples while ST45-MRSA-IV was on the opposite, even though they both found in blood and cerebrospinal fluid sample frequently. Staphylococcal enterotoxin genes *seb*-*seq*-*sek* were strongly associated with ST59 and ST338, while *sec* was associated with ST45, ST121, ST22, and ST30. All ST338, ST1232, and SCC*mec* III isolates carried *lukF/S-PV* genes. A total of 80% of ST338 isolates were resistant to erythromycin, clindamycin, and tetracycline. All ST45 isolates exhibited intermediate or complete resistance to rifampicin. In total, 481 HIS/ASN mutations in *rpoB* were found in rifampicin-resistant or intermediate-resistant isolates. ST338-III and ST45-IV emerged as two of three major clones in MRSA isolates from women and children in Guangzhou, China, though ST59-MRSA-IV remained the most prevalent MRSA clone. Clonal distribution of *S. aureus* varied, depending on the specimen source. Virulence genes and antibiograms were closely associated with the clonal lineage. These results clarified the molecular epidemiology of *S. aureus* from women and children in Guangzhou, China, and provide critical information for the control and treatment of *S. aureus* infections.

## Introduction

*Staphylococcus aureus* is one of the most common pathogens in China, causing a variety of infections, including skin and soft tissue infections (SSTIs), deep-seated abscess, osteoarthritis, necrotizing pneumonia, sepsis, meningitis, and so on (Qiao et al., 2013; Xiao et al., 2015). The continuous and heavy burden of community- and hospital-acquired *S. aureus* infections poses a great threat to public health, especially in children, pregnant women, and postpartum women (Williamson et al., 2014; Liu et al., 2016; Angelopoulou et al., 2018). Recently, a multicenter retrospective cohort study reported that bloodstream infections with methicillin-susceptible (MSSA) and -resistant *S. aureus* (MRSA) increase the daily risk of hospital death and prolonged hospital stay (Stewardson et al., 2016). Unlike in other areas, the prevalence of MRSA in Asia varies substantially among countries and regions; ST239-MRSA-III being prevalent in the north and ST59-MRSA in the south, among pediatric patients in China (Zhang et al., 2009; Chuang and Huang, 2013). Additionally, prevalent MRSA clones have also been changing over the years, from ST239 in 2013 to ST59-MRSA in 2016, among bloodstream isolates across China (Li et al., 2018). The ST45-MRSA-IV clone, also known as the Berlin epidemic strain, either colonizing nares or causing bloodstream infections or respiratory care ward outbreak in Taiwan, is seldom reported in China (Moore et al., 2010; Lee et al., 2011; Monecke et al., 2011; Chow et al., 2017).

Previous studies had indicated that specific virulence genes may play a pivotal role in toxin-mediated diseases, such as enterotoxins B and C (encoded by *seb, sec*) in toxic shock syndrome, enterotoxin A, B, and Q (encoded by *sea, seb*, and *seq*) in food poisoning, and Panton–Valentine leukocidin (PVL, encoded by *lukS-PV* and *lukF-PV* genes) in difficult-to-treat osteomyelitis and severe SSTIs (Karauzum et al., 2012; Spaulding et al., 2013; Hu et al., 2017; Jiang et al., 2017; Nakaminami et al., 2017). Recent studies have demonstrated that another staphylococcal superantigen, SEK (encoded by *sek*), could induce lethal shock in mice (Aguilar et al., 2017). The above virulence genes are encoded by mobile genetic elements (MGEs) in the genome. Though patterns of MGEs are highly variable among *S. aureus* isolates, they are often associated with particular clonal lineages (Lindsay et al., 2006).

*Staphylococcus aureus* developed resistance to different kinds of antimicrobial drugs. It gained resistance to penicillin and methicillin, and gradually co-resistance to methicillin, vancomycin, linezolid, and tigecycline (Kumar, 2016). MRSA emerged from MSSA by the acquisition of the staphylococcal chromosomal cassette *mec* (SCC*mec*). Till date, at least 11 types of SCC*mec* elements have been identified, and SCC*mec* I–V are predominant (Ito et al., 2014). Rifampicin was used as an adjunctive therapy against biofilm or for the treatment of MRSA osteomyelitis (Ashizawa et al., 2016; Yan et al., 2018); however, the burden of rifampicin-resistant and -intermediate *S. aureus* strains in China has been increasing (Xiao et al., 2011). Mutations in *rpoB*, which encodes the β-subunit of RNA polymerase, are closely related to rifampicin resistance (Bongiorno et al., 2018). Since trends in antibiotic susceptibility are changing constantly (Kanjilal et al., 2018; Li et al., 2018), continuous monitoring of prevalence, virulence genes, and antimicrobial resistance patterns of MSSA and MRSA are important for the development and implementation of infection control programs.

## Materials and Methods

### Bacterial Isolates and Data Collection

A total of 131 unduplicated *S. aureus* clinical isolates were obtained between 2015 and 2018 from four centers in Guangzhou, i.e., Zengcheng Maternity and Children’s Health Care Center in Zengcheng district, north of Guangzhou; Nansha Maternity and Children’s Health Care Center in Nansha district, south of Guangzhou; Guangzhou Maternity and Children’s Health Care Center in Yuexiu district, west of Guangzhou, and Guangzhou Women and Children’s Medical Center in Tianhe district, central Guangzhou. *S. aureus* sources were classified into four categories: blood and cerebrospinal fluid (33 isolates from blood and the others 5 from cerebrospinal fluid), pus from women with mastitis (31 isolates), pus from children with SSTIs (21 isolates), and respiratory tract specimens (36 isolates from sputum and the others 5 from alveolar lavage fluid). Information about the isolates was obtained from the laboratory information system. Resistant was defined as a given antibiotic when it was inhibited *in vitro* by a concentration of this drug that is associated with a high likelihood of therapeutic failure while sensitive is associated with a high likelihood of therapeutic success. Intermediate was defined as a given antibiotic when it was inhibited *in vitro* by a concentration of this drug that is associated with an uncertain therapeutic effect (Rodloff et al., 2008). MDR in this study was defined as resistance to three or more non-β-lactam antibiotics (Lee et al., 2017).

### Bacterial Identification and Antibiotic Susceptibility Tests

All *S. aureus* isolates were identified by the automated VITEK2 compact system (bioMérieux, Marcy l’Étoile, France) and detection of *femB* (Jonas et al., 1999). MRSA and MSSA were determined by cefoxitin screening and detection of *mecA*. Susceptibility to 12 antibiotics was analyzed using the automated VITEK2 compact system (bioMérieux, Marcy l’Étoile, France); minimum inhibitory concentration (MIC) interpretive criteria followed the Clinical and Laboratory Standards Institute guidelines (CLSI, 2016). *S. aureus* ATCC 29213 was used as the quality control strain for identification and antibiotic-susceptibility tests.

### DNA Isolation

*Staphylococcus aureus* isolates were cultured and centrifuged as previously described (Liang et al., 2017), and subsequently re-suspended in 300 μl of buffer BS (Takara Bio, Beijing, China). Five microliters of 6 units of lysostaphin was added and incubated at 37°C for 30 min according to the manufacturer’s protocol (Sigma-Aldrich, Shanghai, China). After centrifugation at 13,839 × *g* for 5 min at 4°C, the supernatant was mixed with 200 μl of buffer GB (Takara Bio) and 200 μl of 100% ethanol (Guangzhou Chemical Reagent Factory, Guangzhou, China), and transferred to a spinning tube (Takara Bio). The remaining steps followed the instructions provided with the TaKaRa MiniBEST Bacteria Genomic DNA Extraction Kit Ver.3.0 (Takara Bio).

### Spa Typing

All (*n* = 131) isolates were typed by the spa typing method, according to published protocols (Holtfreter et al., 2007), by amplifying the spa X region. (Primers are listed in Supplementary Table S1). The PCR products were purified and sequenced by a commercial supplier using both amplification primers (Beijing Genomics Institute, Shenzhen, China). Repeats and spa types were assigned using the spa database^1^.

### MLST Typing

All isolates were typed by MLST genotyping, according to a published protocol, by amplifying and sequencing seven housekeeping genes (Enright et al., 2000). STs were confirmed by searching against the MLST database https://pubmlst.org/saureus/. Novel MLST alleles and MLST types were assigned and included in the MLST database. MLST clonal complexes (CCs) were deduced using eBURST v3, based on our own data and a list of distinct ST types with six of the seven loci (Feil et al., 2004). A UPGMA dendrogram was drawn using START2 based on ST types (Jolley et al., 2001).

### SCCmec Analysis

The mec complex of all of the MRSA isolates were typed by multiplex PCR using the primers described by Boye et al. (2007).

### PCR Detection of Virulence Genes

Staphylococcal enterotoxin genes (i.e., *sea, seb, sec, seq*, and *sek*) and the gene encoding Panton–Valentine leukocidin (PVL) were screened by PCR, using all isolates, with the primers shown in Supplementary Table S1 (Lina et al., 1999; Wu et al., 2011; Liang et al., 2017).

### Detection of *rpoB* Mutations

A total of 23 isolates, including 16 rifampicin-resistant or intermediate isolates and 7 rifampicin-sensitive isolates with corresponding sequence types as a control, were amplified by PCR using the primers shown in Supplementary Table S1, yielding an internal gene sequence of 393 bp (from 1257 to 1649), spanning the rifampicin resistance-determining region (Wichelhaus et al., 1999). The PCR products were sequenced by a commercial supplier (Beijing Genomics Institute); the sequences were compared with the full-length *rpoB* sequence from ATCC 29213 (GenBank accession number: LHUS02000145.1) and the NCTC 8325 reference sequence (Gene ID: 3920377) using CLC Sequence Viewer 7 (Qiagen, Düsseldorf, Germany).

### Statistical Analysis

Statistical analyses were performed using SPSS 17.0 Data Editor (SPSS, Inc., Chicago, IL, United States). Categorical variables were described using frequencies and their proportion, and compared using the chi-square (χ^2^) test. *P* < 0.05 was considered to indicate a statistically significant difference.

### Ethics Statement

The current study was approved by the Ethics Committee of Guangzhou Women and Children’s Medical Center (Guangzhou, China; no. 2016081029). Written informed consent was exempted, since this retrospective study mainly focused on bacteria and patient intervention was not required.

## Results

### Bacterial Identification and Antibiotic Susceptibility Tests

Among the 64 MRSA isolates, two were found sensitive, by cefoxitin screening, but carried the *mecA* gene. Antibiotic susceptibility results are summarized in Table 1. The highest rate of resistance was detected for penicillin (PEN) (93.9%), followed by erythromycin (ERY) (58.8%), clindamycin (CLI) (55.7%), tetracycline (TCY) (31.3%), sulfamethoxazole-trimethoprim (SXT) (7.6%), ciprofloxacin (2.3%), rifampicin (1.5%), nitrofurantoin (2.3%), and gentamicin (0.8%). All of the isolates were susceptible to dalfopristin/quinupristin, linezolid, and vancomycin. Rates of resistance to PEN, ERY, CLI, and TCY and intermediate resistance to rifampicin were all significantly higher in the MRSA group than in the MSSA group (*P* < 0.01). In total, 30.5% of isolates were MDR, and the MDR rate for MRSA isolates (50.0%) was significantly higher than that for MSSA (11.9%) (*P* < 0.01).

**Table 1:** Antibiotic profiles of *Staphylococcus aureus* isolated from women and children in Guangzhou, China.

Antibiotic	*S. aureus* (*n* = 131)		MRSA (*n* = 64)		MSSA (*n* = 67)		*P*-value^a^
				
	R, n (%)	I, n (%)	R, n (%)	I, n (%)	R, n (%)	I, n (%)	
Penicillin	123 (93.9)	0 (0.0)	64 (100.0)	0 (0.0)	59 (88.1)	0 (0.0)	<0.01
Erythromycin	77 (58.8)	1 (0.8)	51 (79.7)	0 (0.0)	26 (38.8)	1 (1.5)	<0.01
Clindamycin	73 (55.7)	0 (0.0)	49 (76.6)	0 (0.0)	24 (35.8)	0 (0.0)	<0.01
SXT	10 (7.6)	0 (0.0)	4 (6.3)	0 (0.0)	6 (9.0)	0 (0.0)	0.75
Gentamicin	1 (0.8)	2 (1.5)	0 (0.0)	0 (0.0)	1 (1.5)	2 (3.0)	NA
Vancomycin	0 (0.0)	0 (0.0)	0 (0.0)	0 (0.0)	0 (0.0)	0 (0.0)	NA
Ciprofloxacin	3 (2.3)	2 (1.5)	1 (1.6)	2 (3.1)	2 (3.0)	0 (0.0)	NA
Tetracycline	41 (31.3)	0 (0.0)	30 (46.9)	0 (0.0)	11 (16.4)	0 (0.0)	<0.01
Nitrofurantoin	3 (2.3)	1 (0.8)	2 (3.1)	1 (1.6)	1 (1.5)	0 (0.0)	NA
Rifampicin	2 (1.5)	14 (10.7)	1 (1.6)	11 (17.2)	1 (1.5)	3 (4.5)	0.02^b^
Linezolid	0 (0.0)	0 (0.0)	0 (0.0)	0 (0.0)	0 (0.0)	0 (0.0)	NA
QDA	0 (0.0)	0 (0.0)	0 (0.0)	0 (0.0)	0 (0.0)	0 (0.0)	NA


*MRSA, methicillin-resistant S. aureus; MSSA, methicillin-susceptible S. aureus; SXT, trimethoprim/sulfamethoxazole; QDA, dalfopristin/quinupristin. R, Resistant; I, Intermediate; NA, not applicable. ^*a*^Antibiotic resistance of MRSA vs. MSSA by chi-squared test (two-sided); ^*b*^Rifampicin-intermediate of MRSA vs. MSSA by chi-squared test (two-sided).*

### Overall Molecular Typing

All the isolates were typed by spa and MLST and MRSA isolates (*n* = 64) were subjected to SCCmec typing. In total, 58 spa types, 27 sequence types (STs), and 10 CCs were identified, and 2 spa types were left undetermined. A total of 22 spa types, 12 STs, and 5 CCs were detected in MRSA isolates, whereas 43 spa types, 22 STs, and 10 CCs were detected in MSSA isolates. Three SCC*mec* types were detected and one was undetermined. SCC*mec* types IV and III were predominant, representing 68.8 and 28.1% of isolates, respectively. Seven STs were found in both MRSA and MSSA isolates. Five novel spa types (t17202, t17336, t17651, t17756, and t17757) and five novel STs from isolates with other spa types (ST4512, ST4513, ST4536, ST4538, and ST4553) were identified. The top three spa types among all isolates were t437, t116, and t127, representing 33.6% of isolates. Among MSSA isolates, the top three spa types were t189, t127, and t091, representing 22.4% of isolates. Among MRSA isolates, t437, t116, and t441 represented 62.5% of isolates. The top three STs among all isolates were ST59, ST338, and ST45, representing 39.7% of isolates. Among MSSA isolates, the top three STs were ST188, ST1, and ST398, representing 35.8%, and among MRSA isolates, ST59, ST338, and ST45 represented 78.1% of isolates. The top three CCs among all isolates were CC5, CC59, and CC45, representing 76.3%. Among MSSA isolates, the top three CCs were CC5, CC398, and CC7, representing 79.1% of isolates while CC59, CC45, and CC5 represented 95.3% of MRSA isolates (Table 2).

**Table 2:** Genotype ranking of *S. aureus* isolated from women and children in Guangzhou, China.

Rank	*S. Aureus* (*n* = 131)						MRSA (*n* = 64)						MSSA (*n* = 67)					
			
	SPA		MLST		CCs		SPA		MLST		CCs		SPA		MLST		CCs	
									
		N		N		N		N		N		N		N		N		N
1	t437	28	ST59	24	CC5	44	t437	27	ST59	23	CC59	42	t189	6	ST188	9	CC5	37
2	t116	9	ST338	15	CC59	43	t116	8	ST338	15	CC45	12	t127	5	ST1	8	CC398	10
3	t127	7	ST45	13	CC45	13	t441	5	ST45	12	CC5	7	t091	4	ST398	7	CC7	6
4	t189	6	ST1	13	CC398	10	t3590	3	ST1	5	CC7	2	t034	4	ST7	5	CC121	4
5	t091	5	ST188	9	CC7	8	t127	2	ST7	2	CC88	1	t571	3	ST6	5	CC30	3
Total		55		74		118		45		57		64		22		34		60
%		42.0		56.5		90.1		70.3		89.1		100.0		32.8		50.7		89.6


**FIGURE 1:**
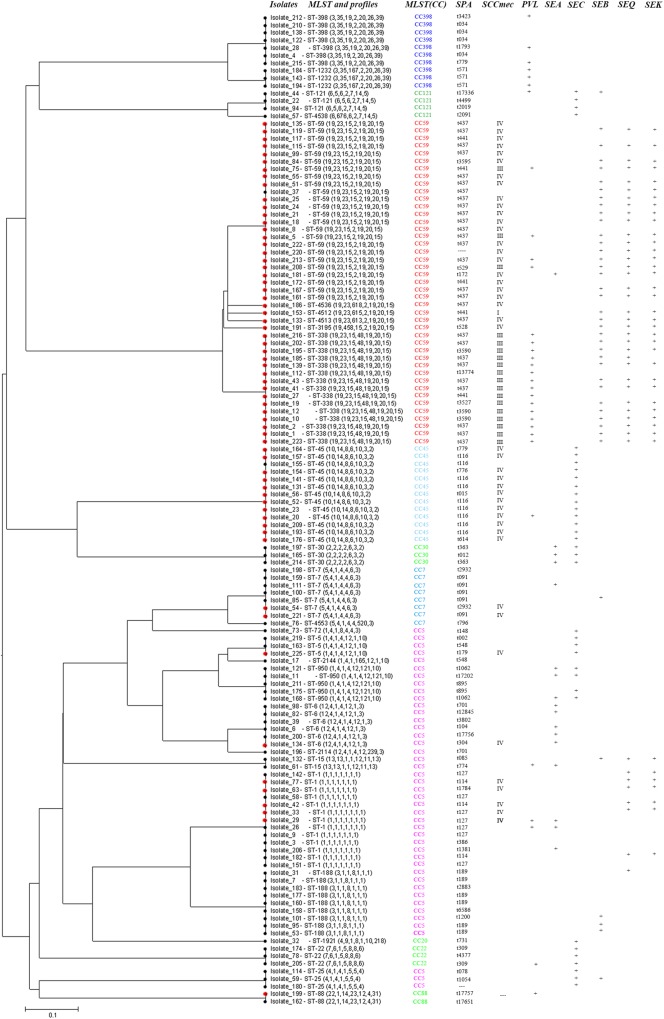
START2 analysis, genotypes, and virulence genes of 131 clinical strains of *Staphylococcus aureus* isolated from women and children in Guangzhou, China.

In pus samples, from women with mastitis and from children, the prevalent STs were ST59, ST338, and ST1, representing 41.9 and 61.9% of isolates, respectively. In the respiratory tract group, the prevalent STs were ST59, ST45, and ST188, accounting for 41.5% of isolates. In the blood and cerebrospinal fluid group, the prevalent STs were ST1, ST59, ST45, ST338, and ST188, accounting for 68.4% of isolates (Table 3). Based on STs, a UPGMA dendrogram was drawn using START2, and isolates formed two major groups; 74.0% of isolates in clade I were MRSA, while 82.8% of isolates in clade II were MSSA (Figure 1).

**Table 3:** Specimen types of *S. aureus* isolated from women and children in Guangzhou, China.

Rank	Specimen types																							
	
	BL and CSF (*n* = 38)						Pus from women with mastitis (*n* = 31)						Pus from SSTIs in children (*n* = 21)						Respiratory tract (*n* = 41)					
				
	SPA		MLST		CCs		SPA		MLST		CCs		SPA		MLST		CCs		SPA		MLST		CCs	
												
		%		%		%		%		%		%		%		%		%		%		%		%
1	t437	21.1	ST1	15.8	CC5	39.5	t437	22.6	ST59	19.4	CC5	35.5	t437	33.3	ST59	28.6	CC59	57.1	t437	14.6	ST59	17.1	CC5	36.6
2	t116	10.5	ST59	13.2	CC59	31.6	t114	6.5	ST338	12.9	CC59	32.3	t114	4.8	ST338	23.8	CC5	14.3	t116	7.3	ST45	14.6	CC59	22.0
3	t189	10.5	ST45	13.2	CC45	13.2	t363	6.5	ST1	9.7	CC398	12.9	t571	4.8	ST1	9.5	CC398	14.3	t091	7.3	ST188	9.8	CC45	14.6
4	t127	10.5	ST338	13.2	CC7	5.3	t571	6.5	ST6	6.5	CC7	6.5	t127	4.8	ST121	9.5	CC121	9.5	t189	4.9	ST7	9.8	CC7	9.8
5	t441	5.3	ST188	13.2	CC121	2.6	t127	3.2	ST1232	6.5	CC30	6.5	t529	4.8	ST398	9.5	CC45	4.8	t441	4.9	ST5	7.3	CC398	4.9
Total		57.9		68.4		92.1		45.2		54.8		93.6		52.4		81.0		100.0		39.0		58.5		87.8


### Specific Sequence Types of *S. aureus* Linked to Certain Virulence Genes and Antibiotic Resistance Patterns

**FIGURE 2:**
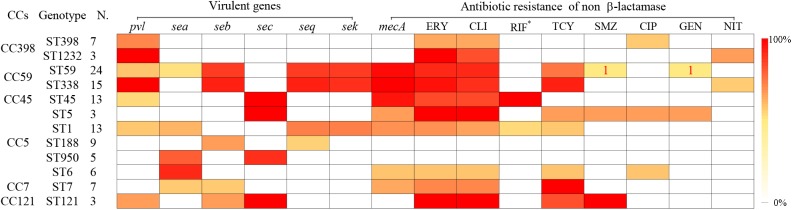
Virulence genes and antigram of *S. aureus* clinical isolates from women and children are linked to STs, as illustrated by the tri-color scale. If a resistant antibiotic occurred in less than 5% of isolates with a particular ST, the number of *S. aureus* isolates resistant to the antibiotic is given. ^∗^Means rifampicin intermediate resistant.

Some specific *S. aureus* STs are strictly linked to certain virulence genes and antibiotic resistance patterns, though they are located in MGEs (Figure 2). For example, all SCC*mec* type III isolates carried *lukS/F-PV* genes encoding Panton–Valentine leukocidin (PVL). All ST338 isolates carried *lukS/F-PV* genes and more than 80% carried *seb, seq*, and *sek* and were resistant to ERY, CLI, and TCY with an MDR rate of 80%. ST59 and ST338 isolates belonged to the same CC, exhibited similar frequencies of *seb, seq*, and *sek* genes, and had similar rates of resistance to ERY and CLI antibiotics, with an MDR rate of 54.2%. However, only 16.7% carried *lukS/F-PV* genes. All ST45 isolates carried the *sec* gene and exhibited intermediate resistance or were resistant to RIF, but none carried *sea, seb, seq*, or *sek* genes or was resistant to TCY, which was otherwise very common among CC59 isolates. ST45 isolates had a low MDR rate of 7.7%. All of the ST30, ST22, and ST121 isolates possessed the *sec* gene, and the latter isolates were also resistant to ERY, CLI, and SXT antibiotics with an MDR rate of 100%. All ST1232 isolates carried *lukS/F-PV* genes and were resistant to ERY. All ST7 isolates were resistant to TCY with an MDR rate of 42.9%. The carriage of *lukS/F-PV* genes was more common among MRSA isolates (34.4%) than among MSSA isolates (14.9%) (*P* = 0.01).

### RIF Resistant or Intermediate Isolates and *rpoB* Mutations

A total of 23 isolates, including 16 rifampicin-resistant or intermediate isolates and 7 rifampicin-sensitive isolates with the corresponding sequence types as a control, were detected. In total, 93.8% of the rifampicin-resistant or intermediate isolates belonged to ST45. Compared to the full-length *rpoB* sequence from ATCC 29213 and NCTC 8325, four nucleotide mutations were identified at positions 1422, 1441, 1506, and 1533. However, only one amino acid substitution was found at position 481, i.e., a change from HIS to ASN, among all the 16 rifampicin-resistant or intermediate isolates; amino acid substitutions were not detected among the 7 rifampicin-sensitive isolates.

## Discussion

*Staphylococcus aureus* is one of the most common pathogens in China, causing a variety of infections. Its population structure and antibiotic susceptibility are changing constantly (Kanjilal et al., 2018; Li et al., 2018). In this study, we investigated the prevalence, virulence genes, and antimicrobial resistance patterns of 131 isolates (67 MSSA and 64 MRSA) collected from four centers for women and children in Guangzhou, southern China.

Both the number of spa types and MLSTs were greater in the MSSA group than in the MRSA group, suggesting that the MSSA isolates were more diverse than MRSA isolates. Of the 27 STs, only 7 ST genotypes were shared between MRSA and MSSA isolates, and 74.0% of isolates assigned to clade I were MRSA while 82.8% isolates in clade II were MSSA, hence suggesting that the groups evolved in a relatively independent manner, except for the gain and loss of methicillin resistance among some epidemic clones (Ledda et al., 2017).

The epidemiology of prevalent MRSA clones is changing dynamically and geographically. Previous studies had reported high prevalence of ST59 and ST338 in community-acquired infections across China while ST8 was the major clonal lineage in United States and Europe (Chuang and Huang, 2013; Carrel et al., 2015). In this study, on women and children in Guangzhou, southern China, ST8-MRSA strain was not found, like most other studies in China (Wang et al., 2012; Wang X. et al., 2018). ST59-MRSA-IV remained the major MRSA clone, accounting for 31.3% of isolates. Proportion of ST338-MRSA-III clones accounted for 36.6% of CC59 isolates in this study, higher than 9% of CC59 CA-MRSA isolates obtained from children in seven major cities in China, reported previously (Wang et al., 2012). Compared to ST59-MRSA clone, all ST338-MRSA-III isolates carried *lukS/F-PV* genes, although both of them had similar antimicrobial resistance patterns (ERY-CLI-TCY). In addition, it was mostly found in pus and blood, but hardly in the respiratory tract samples.

Contrary to ST59 and ST338, ST45-MRSA clone was mostly found in the respiratory tract and blood, but hardly found in pus samples. This may possibly be due to the lack of *lukS/F-PV* genes. It exhibited different antimicrobial resistance patterns (ERY-CLI-RIF), although most of the strains showed intermediate resistance to RIF. One amino acid substitution was found at position 481 (a change from HIS to ASN) in rpoB protein. In this study, ST45 ranked third among the MRSA isolates, accounting for 18.8%, compared to 1.7% in MRSA isolates from Chinese children in a previous report (Wang et al., 2012). ST338-MRSA-III and ST45-MRSA-IV emerged as two major MRSA clones, suggesting changing clonal structure of MRSA in this region of China. As a result of low levels of ciprofloxacin and gentamicin resistance among the three major MRSA clones in this study, the overall resistant levels were low. These results were differing from previous study from other regions where ST239 or ST5 MRSA clones dominated, the antimicrobial resistance profiles of which were often ciprofloxacin, gentamicin resistant (Cheng et al., 2013), hence their resistant levels would be much higher.

In this study, the most frequent STs for MSSA were ST188 (13.4%), ST1 (11.9%), and ST398 (10.4%). ST188, with strong biofilm-formation and adhesion ability, remained the most common MSSA clones in infections among children in China; however, the proportion of ST1 MSSA isolates was higher in this study than in previous estimates, especially in blood and CSF group (Wang Y. et al., 2018). It may probably be because ST1 was the most common ST recently in retail ready-to-eat food and aquatic products across China (Rong et al., 2017; Yang et al., 2018). Human-adapted ST398 MSSA isolates, belonging to clade I, often cause severe and fatal infections (Uhlemann et al., 2012; Zhao et al., 2012). In this study, 37.5% of the isolates possessed *lukS/F-PV* genes. ST1232, a single locus variant of ST398, was first reported to cause SSTIs in humans. All the ST1232 isolates carried *lukS/PV* genes and were resistant to ERY.

Methicillin-susceptible *S. aureus* could be transmitted to preterm infants through breastfeeding (Kayiran et al., 2014), since breast milk is a reservoir for *S. aureus* (Li et al., 2017). However, in China, little is known about the prevalence of *S. aureus* in women with mastitis, especially during the lactation period. In this study, we analyzed pus specimens from women with mastitis (67.7% during the lactation period) and pus from children with SSTIs, and found the clonal structures to be similar, except for the higher proportion of CC59 in the latter group, thereby suggesting a risk of cross-infection through breastfeeding and close contact between mother and baby.

Limitations of the present study include the small number of isolates assigned to each group and the potential bias that may exist on the selection of samples. Two isolates, carrying the *mecA* gene, namely OS-MRSA, were found to be sensitive by cefoxitin screening. One of them was scc*mec* type III while the other was not classified by scc*mec* typing; whole genome sequencing should be applied to analyze the underlying mechanism.

In summary, our results showed that MSSA and MRSA evolved in a relatively independent manner; ST338-MRSA-III and ST45-MRSA-IV have emerged as two of three major clones in Guangzhou, China, though ST59-MRSA-IV remained the most prevalent MRSA clone. The clonal distribution of *S. aureus* isolates from women and children differed among specimen sources. Virulence genes and antimicrobial resistance patterns were closely associated with some specific clonal lineages. Our study provides updated data related to the molecular epidemiology of *S. aureus* from women and children in Guangzhou, China.

## Author Contributions

ZZ and HY conceived and designed the study. BL, JM, YuL, YiH, HZ, YX, QD, and LH performed the experiments described in this study. SY, YgH, and YaL analyzed the data. BL wrote the draft. YY, SG, ZZ, and HY revised the manuscript. All authors have approved the final version.

## Conflict of Interest Statement

The authors declare that the research was conducted in the absence of any commercial or financial relationships that could be construed as a potential conflict of interest.

## Footnotes

1 https://www.spaserver.ridom.de
