# Supplementary material for: Prevalence and Characterization of Staphylococcus aureus Isolated From Women and Children in Guangzhou, China
Source: Front Microbiol. 2018 Nov 16;9:2790. doi: 10.3389/fmicb.2018.02790 (PMC6250813; doi:10.3389/fmicb.2018.02790)
Supplement: Supplementary file 1 [file Table_1.DOCX]

Table S1 Primers used for multiplex and singleplex PCRs

| PCR | Name | Gene | Primer sequence (5‘‐3‘) | Primer | Product | Reference |
| --- | --- | --- | --- | --- | --- | --- |
|  |  |  |  | size (bp) | size (bp) |  |
| 1 | FemB-f | femB | TTACAGAGTTAACTGTTACC | 20 | 651 | 1 |
|  | FemB-r |  | ATACAAATCCAGCACGCTCT | 20 |  |  |
|  | MecA-f | mecA | GTAGAAATGACTGAACGTCCGATAA | 25 | 310 | 1 |
|  | MecA-r |  | CCAATTCCACATTGTTTCGGTCTAA | 25 |  |  |
| 2 | spa-1113f | spa | TAAAGACGATCCTTCGGTGAGC | 22 | variable | 2 |
|  | spa-1514r |  | CAGCAGTAGTGCCGTTTGCTT | 21 | variable |  |
| 3 | arc up | arc | TTGATTCACCAGCGCGTATTGTC | 23 | 456 | 3 |
|  | arc dn |  | AGGTATCTGCTTCAATCAGCG | 21 |  |  |
| 4 | aro up | aro | ATCGGAAATCCTATTTCACATTC | 23 | 456 |  |
|  | aro dn |  | GGTGTTGTATTAATAACGATATC | 23 |  |  |
| 5 | glp up | glp | CTAGGAACTGCAATCTTAATCC | 22 | 465 |  |
|  | glp dn |  | TGGTAAAATCGCATGTCCAATTC | 23 |  |  |
| 6 | gmk up | gmk | ATCGTTTTATCGGGACCATC | 20 | 429 |  |
|  | gmk dn |  | TCATTAACTACAACGTAATCGTA | 23 |  |  |
| 7 | pta up | pta | GTTAAAATCGTATTACCTGAAGG | 23 | 474 |  |
|  | pta dn |  | GACCCTTTTGTTGAAAAGCTTAA | 23 |  |  |
| 8 | tpi up | tpi | TCGTTCATTCTGAACGTCGTGAA | 23 | 402 |  |
|  | tpi dn |  | TTTGCACCTTCTAACAATTGTAC | 23 |  |  |
| 9 | yqi up | yqi | CAGCATACAGGACACCTATTGGC | 23 | 516 |  |
|  | yqi dn |  | CGTTGAGGAATCGATACTGGAAC | 23 |  |  |
| 10 | b | ccrA2-B | ATTGCCTTGATAATAGCCYTCT | 22 | 937 | 4 |
|  | a3 |  | TAAAGGCATCAATGCACAAACACT | 24 |  |  |
|  | ccrC1 | ccrC | CGTCTATTACAAGATGTTAAGGATAAT | 27 | 518 |  |
|  | ccrC2 |  | CCTTTATAGACTGGATTATTCAAAATAT | 28 |  |  |
|  | 1272F | IS1272 | GCCACTCATAACATATGGAA | 20 | 415 |  |
|  | 1272R |  | CATCCGAGTGAAACCCAAA | 19 |  |  |
|  | 5RmecA | mecA–IS431 | TATACCAAACCCGACAACTAC | 21 | 359 |  |
|  | 5R431 |  | CGGCTACAGTGATAACATCC | 20 |  |  |
| 11 | luk-PV-1 | pvl | ATCATTAGGTAAAATGTCTGGACATGATCCA | 31 | 433 | 5 |
|  | luk-PV-2 |  | GCATCAASTGTATTGGATAGCAAAAGC | 27 |  |  |
| 12 | sea-f | sea | GAAAAAAGTCTGAATTGCAGGGAACA | 26 | 560 | 6 |
|  | sea-r |  | CAAATAAATCGTAATTAACCGAAGGTTC | 28 |  |  |
| 13 | seb-f | seb | ATTCTATTAAGGACACTAAGTTAGGGA | 27 | 404 |  |
|  | seb-r |  | ATCCCGTTTCATAAGGCGAGT | 21 |  |  |
| 14 | sec-f | sec | ggcGAGCTCATGAAGTTATTTGCTTTTATCTTC | 33 | 771 | This work |
|  | sec-r |  | gcgCTCGAGTTATTTTTTGGTTAAATGAACTTC | 33 |  |  |
| 15 | seq-f | seq | GCAGTCGACATGCCTATATGGCGTTGTAATATA | 33 | 771 | 7  This work |
|  | seq-r |  | CCGCTCGAGTTATTCAGTTTTCTCATATGAAATC | 34 |  |  |
| 16  17 | sek-f | sek  rpoB | GCCGTCGACATGAAAAAATTAATAAGCATCTTATTA | 36 | 729  393 |  |
|  | sek-r  rpoB-f  rpoB-r |  | CCGCTCGAGTTATATCGTTTCTTTATAAGAAATATCG  CCGTATCGGTTTATCAAGAATG  TCAACTTTACGATATGGTGTTT | 37  22  22 |  |  |

Reference:

1. Jonas D, Grundmann H, Hartung D, Daschner FD, Towner KJ. Evaluation of the mecA femB duplex polymerase chain reaction for detection of methicillin-resistant Staphylococcus aureus. European journal of clinical microbiology & infectious diseases: official publication of the European Society of Clinical Microbiology (1999) 18(9):643-7. PubMed PMID: 10534186.

2. http://www.ridom.de/doc/Ridom_spa_sequencing.pdf

3. http://saureus.mlst.net/misc/info.asp#experimental

4. Boye K, Bartels MD, Andersen IS, Moller JA, Westh H. A new multiplex PCR for easy screening of methicillin-resistant Staphylococcus aureus SCCmec types I-V. Clinical microbiology and infection : the official publication of the European Society of Clinical Microbiology and Infectious Diseases (2007) 13(7):725-7. doi: 10.1111/j.1469-0691.2007.01720.x. PubMed PMID: 17403127.5 Gerard Lina Clinical Infectious Diseases

6. Wu D, Li X, Yang Y, Zheng Y, Wang C, Deng L, et al. Superantigen gene profiles and presence of exfoliative toxin genes in community-acquired meticillin-resistant Staphylococcus aureus isolated from Chinese children. Journal of medical microbiology (2011) 60(Pt 1):35-45. doi: 10.1099/jmm.0.023465-0. PubMed PMID: 20829395.

7. Liang BS, Huang YM, Chen YS, Dong H, Mai JL, Xie YQ, et al. Antimicrobial resistance and prevalence of CvfB, SEK and SEQ genes among Staphylococcus aureus isolates from paediatric patients with bloodstream infections. Experimental and therapeutic medicine (2017) 14(5):5143-8. doi: 10.3892/etm.2017.5199. PubMed PMID: 29201229; PubMed Central PMCID: PMC5704349.
